# Ensemble stacking rockburst prediction model based on Yeo–Johnson, K-means SMOTE, and optimal rockburst feature dimension determination

**DOI:** 10.1038/s41598-022-19669-5

**Published:** 2022-09-12

**Authors:** Lijun Sun, Nanyan Hu, Yicheng Ye, Wenkan Tan, Menglong Wu, Xianhua Wang, Zhaoyun Huang

**Affiliations:** 1 School of Resources and Environmental Engineering, Wuhan University of Science and Technology, Wuhan, 430081 Hubei China; 2 Wuhan Safety and Environmental Protection Research Institute of Sinosteel Group, Wuhan, 430081 Hubei China; 3 Hubei Jingshen Safety Technology Co., Ltd., Yichang, 443000 Hubei China

**Keywords:** Engineering, Civil engineering

## Abstract

Rockburst forecasting plays a crucial role in prevention and control of rockburst disaster. To improve the accuracy of rockburst prediction at the data structure and algorithm levels, the Yeo–Johnson transform, K-means SMOTE oversampling, and optimal rockburst feature dimension determination are used to optimize the data structure. At the algorithm optimization level, ensemble stacking rockburst prediction is performed based on the data structure optimization. First, to solve the problem of many outliers and data imbalance in the distribution of rockburst data, the Yeo–Johnson transform and k-means SMOTE algorithm are respectively used to solve the problems. Then, based on six original rockburst features, 21 new features are generated using the PolynomialFeatures function in Sklearn. Principal component analysis (PCA) dimensionality reduction is applied to eliminate the correlations between the 27 features. Thirteen types of machine learning algorithms are used to predict datasets that retain different numbers of features after dimensionality reduction to determine the optimal rockburst feature dimension. Finally, the 14-feature rockburst dataset is used as the input for integrated stacking. The results show that the ensemble stacking model based on Yeo–Johnson, K-means SMOTE, and optimal rockburst feature dimension determination can improve the accuracy of rockburst prediction by 0.1602–0.3636. Compared with the 13 single machine learning models without data preprocessing, this data structure optimization and algorithm optimization method effectively improves the accuracy of rockburst prediction.

## Introduction

Rockbursts have always been a difficult and important topic in rock mechanics research. Rockbursts are frequent geological disasters in the construction of water conservancy, hydropower, and transportation projects; deep mining; geological treatment of nuclear waste; and deep physical underground laboratories^1,2^. They not only affect the construction process but also threaten lives and property^3,4^. If the occurrence of a rockburst can be accurately predicted and protective measures taken in advance to reduce the occurrence of rockbursts, staff casualties and property losses can be greatly reduced. Therefore, rockburst prediction has attracted much attention in the past two decades^5^.

There are four categories of rockburst prediction methods: empirical methods^6–8^, simulation techniques^9–11^, mathematical algorithms^12–14^, and monitoring technologies^15–17^. Both empirical methods and simulation techniques use similar simulation tests, and there is a certain gap between the rockburst phenomenon in the laboratory and the actual engineering. Monitoring technologies it is difficult to determine the threshold of rockburst, and the monitoring equipment is easily damaged during underground mining. With the advent of big data and artificial intelligence, rockburst prediction research methods based on mathematical algorithms have become increasingly prominent. Initially, a single model was used for rockburst prediction. For example, Feng et al.^18^ built a support vector machine model, Zhou et al.^19^ built a fisher discriminant analysis model, and Dong et al.^20^ built a random forest model for rockburst prediction. With further development of these techniques, combinations of multiple algorithms have been used to improve the prediction ability at the algorithm level. The use of multiple algorithms can compensate for the difficulty of determining the optimal parameters for a single model, the difficulty of determining the weights of rockburst influencing factors, and the subjectivity of the weights of rockburst influencing factors. On this basis, rockburst prediction models were optimized to improve their rockburst prediction ability. Zhu et al.^21^ Established a rockburst prediction method based on improved support vector machine algorithm (SVR). Zhou et al.^22^ applied the rough set (RS) theory to calculate the support and weight of each rockburst index, which was combined with the approximate ideal solution sequencing method (TOPSIS) to determine the rockburst grade. The established RS-TOPSIS method was applied for rockburst prediction in deep mines and metal mines. Peng et al.^23^ used a real-coded GA to select the optimal support vector machine (SVM) model parameters and established a GA-SVM rockburst prediction model.

Currently, there is a comprehensive effort to improve the rockburst prediction ability at the data structure and algorithm level. Different methods have been used to solve the problems of outliers, missing values, and data imbalances in rockburst datasets. The concept of an ensemble model combines multiple machine learning algorithms to obtain a model with stronger learning capabilities. For example, Zhang et al.^24^ used nine data interpolation algorithms to estimate the missing values in a rockburst dataset and aggregated seven individual machine learning algorithms. Yin et al.^25^ combined three data mining techniques: principal component analysis, local outlier factors, and an expectation maximization algorithm for dimension reduction, outlier detection, and outlier substitution, respectively. Ensemble stacking technology integrates the K-nearest neighbor (KNN), SVM, deep neural network (DNN), and recurrent neural network (RNN) methods. Wang et al.^26^ developed bagging and boosting tree-based ensemble techniques. Of these, bagging was the best method for rockburst prediction.

In summary, many scholars have proposed new methods for rockburst prediction based on mathematical algorithms from both the data structure and algorithm levels^27,28^. At the data structure level, to address missing values and outliers in the rockburst dataset, the data interpolation method has been used to supplement the missing values, and the local outlier factor (LOF) algorithm has been used to detect, replace, and eliminate outliers^29^. However, the estimation of missing values and replacement or elimination of outliers may destroy the original characteristics of the dataset. It also ignores a small number of objective laws. Oversampling is usually used to solve the problem of rockburst data imbalance, and the synthetic minority over-sampling technique (SMOTE), Boderline-SMOTE1, Boderline-SMOTE2, and SMOTE-NC algorithms have all been used previously to solve the problem of within-class imbalance. However, the problem of regional between-class imbalance has not been solved. In terms of the dimensions of rockburst features, the principal component analysis (PCA) algorithm is generally used to reduce the dimensionality of rockburst features. However, ignoring a number of existing rockburst features in the rockburst dataset may result in a dataset that is not sufficient to obtain optimal performance of the model. Hence, increasing the feature dimensions appropriately can improve the prediction ability of the model. At the algorithm level, the ensemble method has advantages over the use of single machine learning algorithms. There are three types of ensemble methods: stacking, bagging, and boosting. They are all considered in this study.

Therefore, this study aims to approach the limit state of rockburst prediction. A total of 275 sets of rockburst case samples with no missing values are collected from the literature. At the data structure level, the Yeo–Johnson transformation, K-means SMOTE balance processing, rockburst feature analysis, and optimal rockburst feature dimension determination are performed on the rockburst data. At the algorithm level, ensemble stacking is used for rockburst prediction to obtain the best accuracy of the rockburst classification prediction. To verify the effectiveness of the data structure optimization, the prediction results of 13 machine learning algorithms are used for comparison before and after data structure optimization. The 13 machine learning algorithms include models with both poor learning performance and strong learning performance.

The rockburst dataset transformation and balancing are described in section “Yeo–Johnson transformation and balancing of the rockburst data”. To deal with outliers in the rockburst dataset, Yeo–Johnson transform is used to normalize the data, reduce the distance between outliers and dense area points, and reduce the influence of outliers. To address the problem of unbalanced rockburst data, the K-means SMOTE algorithm is used to oversample the rockburst dataset after the Yeo–Johnson transformation. This balances the rockburst data. The Yeo–Johnson transformation method can reduce the heteroscedasticity caused by the rockburst data originating from different regions or engineering backgrounds, and amplify the normality of the rockburst data. The degree of outliers in the rockburst data is reduced, and the number of outliers in the rockburst data is reduced. The K-means SMOTE oversampling method is a combination of the K-means clustering algorithm and the SMOTE oversampling algorithm. First, the rockburst data is divided into k clusters by the K-means algorithm, and then the clusters with a high proportion of minority samples are filtered out, and finally the clusters with a high proportion of minority samples are subjected to SMOTE oversampling. This method adds a small number of grade I samples, grade II samples, and grade IV samples to keep the number basically the same as the number of grade III samples, and balances the number of rockburst samples of different grades. To verify the validity of the Yeo–Johnson transformation and the K-means SMOTE balancing process, 13 machine learning algorithms are used to analyze the original rockburst dataset and the rockburst dataset subjected to the Yeo–Johnson transformation and K-means SMOTE oversampling. The prediction results are compared and analyzed to verify the effectiveness of the preprocessing method.

The rockburst feature analysis and determination of the optimal rockburst feature dimensions are presented in section “Rockburst data feature analysis and determination of the optimal rockburst feature dimensions”. First, the mean decrease accuracy graph and Pearson correlation coefficient figures are used to analyze the rockburst features. It is determined that all of the rockburst features are predictive to some extent, and the features are not completely correlated. Then, based on the six original rockburst features, 21 new rockburst features are generated to increase the feature dimension. PCA dimensionality reduction is applied to process the 27 rockburst features, eliminate the correlations between rockburst features, and ensure the features are independent. Finally, the exhaustive method is used to identify the number of features that provides the highest average accuracy under the 13 machine learning algorithms.

Rockburst prediction based on stacking is presented in section “Rockburst prediction based on ensemble stacking”. A rockburst dataset comprising 14 features is input for stacking. Then, an appropriate model is selected from the 13 machine learning algorithms as the first layer base model for stacking. The logistic regression classifier is used as the second output model for stacking, which obtains the rockburst prediction results. The XgBoost model, which has the highest learning ability for the rockburst dataset comprising 14 features, is compared with the ensemble stacking model. The results demonstrate the advantages of the ensemble stacking model.

## Rockburst data acquisition and analysis

Although there are numerous records of rockburst cases around the world, the impact factors of rockbursts in related cases are very limited. A total of 275 rockburst case samples with no duplicates or missing values were collected from the literature. The overall sample includes 51 groups of no rockburst occurrence (I) samples, 74 groups of weak rockburst (II) samples, 117 groups of moderate rockburst (III) samples, and 33 groups of strong rockburst (IV) samples. Based on previous research in machine learning and a comprehensive evaluation of rockburst influencing factors used in rockburst prediction, the following were selected as the rockburst prediction features: the maximum tangential stress of the surrounding rock ($\sigma_{\theta }$), uniaxial compressive strength of the rock ($\sigma_{c}$), uniaxial tensile strength of the rock ($\sigma_{t}$), rock elastic strain energy index ($W_{et}$), rock stress coefficient *SR*($\sigma_{\theta } /\sigma_{c}$)and rock brittleness coefficient *BR*($\sigma_{c} /\sigma_{t}$).

**Figure 1:**
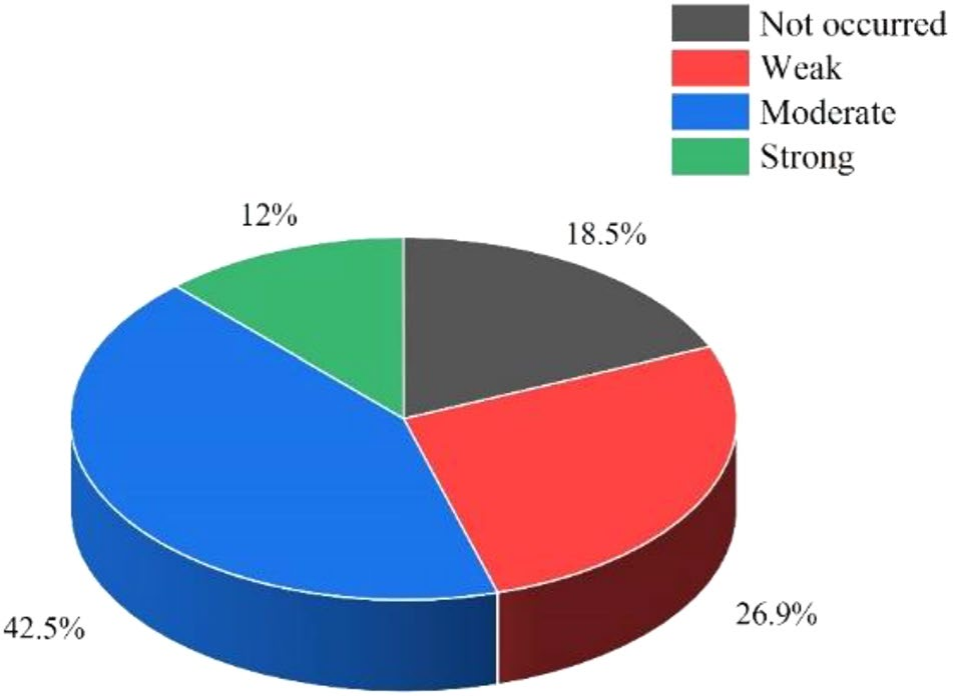
Proportion of each rockburst grade in the dataset.

**Figure 2:**
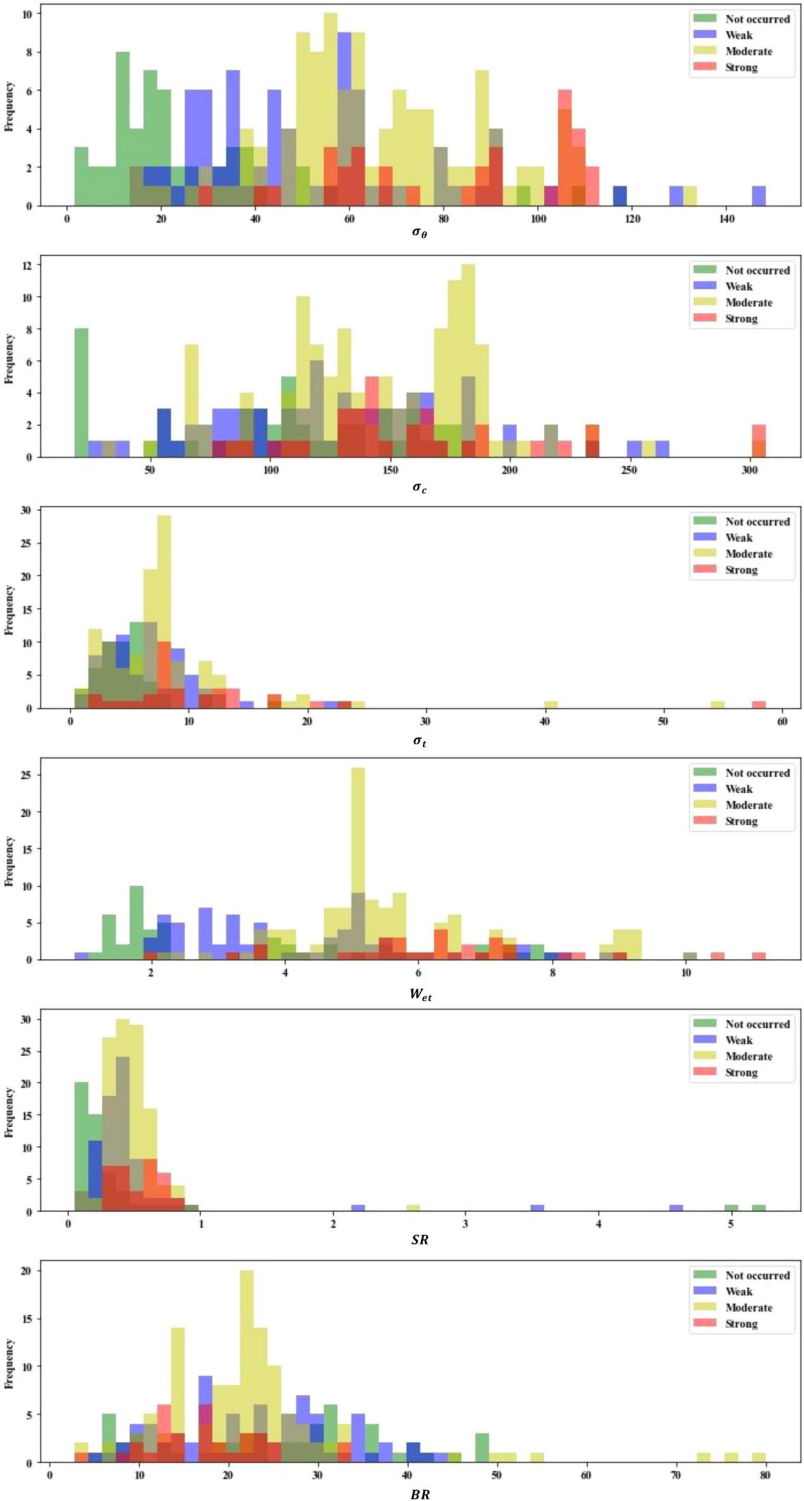
Overlaid histograms of each feature in the rockburst dataset.

A comprehensive understanding of the rockburst dataset characteristics is a prerequisite for data structure optimization. Therefore, the statistical parameters of the features for each rockburst grade are listed in Table 1. The relevant rockburst classification standards^30–32^ are summarized in Table 2, which combines domestic and international criteria and engineering cases of rockbursts as well as the classification standard for the Qinling Tunnel in China and the classification standard for rockbursts suggested by the Ministry of Railways. Figure 1 shows the proportion of each grade of rockburst, and Fig. 2 shows the overlaid histograms of each feature in the rockburst dataset.

**Table 1:** Statistical parameters of different rockburst grades.

Rockburst grades	Rockburst features						
	Statistical parameters	$\sigma_{\theta }$ (MPa)	$\sigma_{c}$ (MPa)	$\sigma_{t}$ (MPa)	$W_{et}$	*SR*	*BR*
I	Maximum	118.40	237.10	17.66	7.90	5.26	8.21
	Minimum	1.60	18.32	0.38	1.10	0.05	45.42
	Mean	26.38	104.30	4.83	3.43	0.43	24.81
	Coefficient of variation	0.90	0.51	0.58	0.62	2.26	0.50
II	Maximum	148.40	263.00	22.60	10.00	4.55	42.96
	Minimum	13.50	26.06	0.77	0.85	0.11	4.48
	Mean	51.83	127.88	6.71	4.31	0.51	22.94
	Coefficient of variation	0.52	0.39	0.61	0.44	1.27	0.41
III	Maximum	132.10	304.00	54.15	10.00	2.56	80.00
	Minimum	14.40	30.00	1.50	2.03	0.09	2.97
	Mean	65.52	145.75	8.21	5.52	0.48	23.00
	Coefficient of variation	0.34	0.31	0.82	0.28	0.5	0.52
IV	Maximum	110.35	306.58	58.59	11.20	0.82	32.24
	Minimum	30.10	80.60	2.50	1.90	0.26	2.80
	Mean	82.37	160.34	11.50	6.34	0.54	17.58
	Coefficient of variation	0.29	0.34	0.83	0.29	0.30	0.37

**Table 2:** Rockburst classification standards.

Rockburst grades	Rockburst features					
	$\sigma_{\theta }$ (MPa)	$\sigma_{c}$ (MPa)	$\sigma_{t}$ (MPa)	$W_{et}$	*SR*	*BR*
I	0–24.0	0–80.0	0–5.0	0–2.0	0.1–0.3	40.0–53.0
II	24.0–60.0	80.0–120.0	5.0–7.0	2.0–3.5	0.3–0.5	26.7–40.0
III	60.0–126.0	120.0–180.0	7.0–9.0	3.5–5.0	0.5–0.7	14.5–26.7
IV	126.0–200.0	180.0–320.0	9.0–30.0	5.0–6.5	0.7–0.9	0–14.5

In Fig. 1, the ratio of rockburst samples of grades I, II, III, and IV is 1.8:2.7:4.3:1.2; thus, the numbers of rockburst samples in each grade are unbalanced. In particular, the number of grade IV and grade III samples is quite different, and the ratio of grade IV samples to grade III samples is 1:3.5. We assume that there are only grade IV samples and grade III samples in the rockburst dataset. When all grade IV samples are predicted as grade III samples, the prediction accuracy rate for grade III samples can reach 78%. Therefore, in the unbalanced rockburst dataset, the classification results are often affected by the majority class. Machine learning models can easily divide minority class samples into majority class samples^33^.

It can be observed from the rockburst classification standards in Table 2, that as the values of features $\sigma_{\theta }$, $\sigma_{c}$, $\sigma_{t}$, $W_{et}$, and *SR* increase, the rockburst grade increases accordingly. In contrast, the rockburst grade increases as the value of feature *BR* decreases. However, the average value of *SR* in grade II (0.51) is greater than that for grade III (0.48), while the average value of *BR* in grade II (22.94) is less than that in grade III (0.48). This is inconsistent with the increasing and decreasing trends of the rockburst classification standards. The reason for this phenomenon is that the maximum and minimum values of the features for each rockburst grade are quite different, the coefficients of variation of the rockburst features are large, and there are many outliers. As shown in Fig. 2, there are many outliers for each feature for different rockburst grades, and the sparse outliers are far away from the dense area of points. Thus, it is difficult to distinguish the rockburst grade based only on the value of a single feature. According to the rockburst classification standard in Table 2, single features were used to judge the rockburst grades of the dataset. The accuracy rates of $\sigma_{\theta }$, $\sigma_{c}$, $\sigma_{t}$, $W_{et}$, *SR*, and *BR* were 0.48, 0.35, 0.35, 0.33, 0.39, and 0.44, respectively. Therefore, the single features have low accuracy for judging the grade of rockburst cases. The root cause of this phenomenon is the failure of rockburst grades to fully reflect the influence of rockburst control factors. Therefore, comprehensively judging the grades of rockburst cases based on multiple features can provide higher accuracy.

## Yeo–Johnson transformation and balancing of the rockburst data

### Yeo–Johnson transformation

The preprocessing of the rockburst dataset mainly solves the problems of a large number of outliers in the dataset and the imbalance in the number of samples of each rockburst grade. For outliers in the dataset. Tan et al.^29^ proposed the LOF algorithm to detect and remove outliers in a dataset. Yin et al.^25^ proposed the LOF algorithm to detect outliers in a dataset and replaced them using the expectation maximization (EM) algorithm. Properly removing or replacing outliers in the spatial distribution of rockburst samples can effectively improve the data structure and the rockburst prediction ability. However, outliers in the rockburst dataset are inherent attributes. The elimination and replacement of outliers may destroy the original characteristics of the data and ignore a small number of objective laws.

In view of this, the Yeo–Johnson^34^ transform was proposed to process the rockburst features. This method is a power transformation, which is often used in the data preprocessing stage of data mining and machine learning. It can reduce the heteroscedasticity of rockburst features and amplify the normality, thus resulting in a probability density function that is closer to a normal distribution. Compared with directly removing or replacing outliers, the Yeo–Johnson transformation retains outliers in the original dataset, improves the data structure, and reduces the influence of outliers on the prediction results.

The Yeo–Johnson transformation is defined as follows:

1 $$ \psi (\lambda ,y) = \left\{ {\begin{array}{*{20}l} {\{ (y + 1)^{\lambda } - 1\} /\lambda } & {\quad if\;\lambda \ne 0,\;y \ge 0} \\ {\log (y + 1)} & {\quad if\;\lambda = 0,\;y \ge 0} \\ { - \{ ( - y + 1)^{2 - \lambda } - 1\} /(2 - \lambda )} & {\quad if\;\lambda \ne 2,\;y < 0} \\ { - \log ( - y + 1)} & {\quad if\;\lambda = 2,\;y < 0} \\ \end{array} } \right. $$

where *y* is the rockburst feature data, and $\lambda$ is the parameter estimated by the maximum likelihood method.

**Figure 3:**
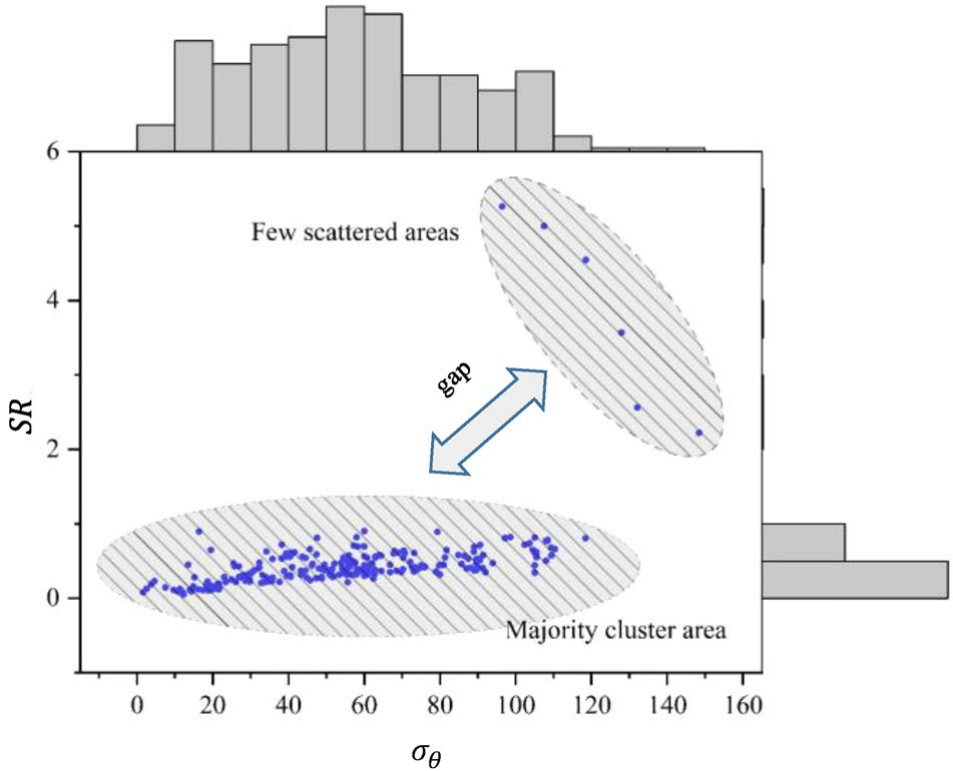
Unscaled data features.

**Figure 4:**
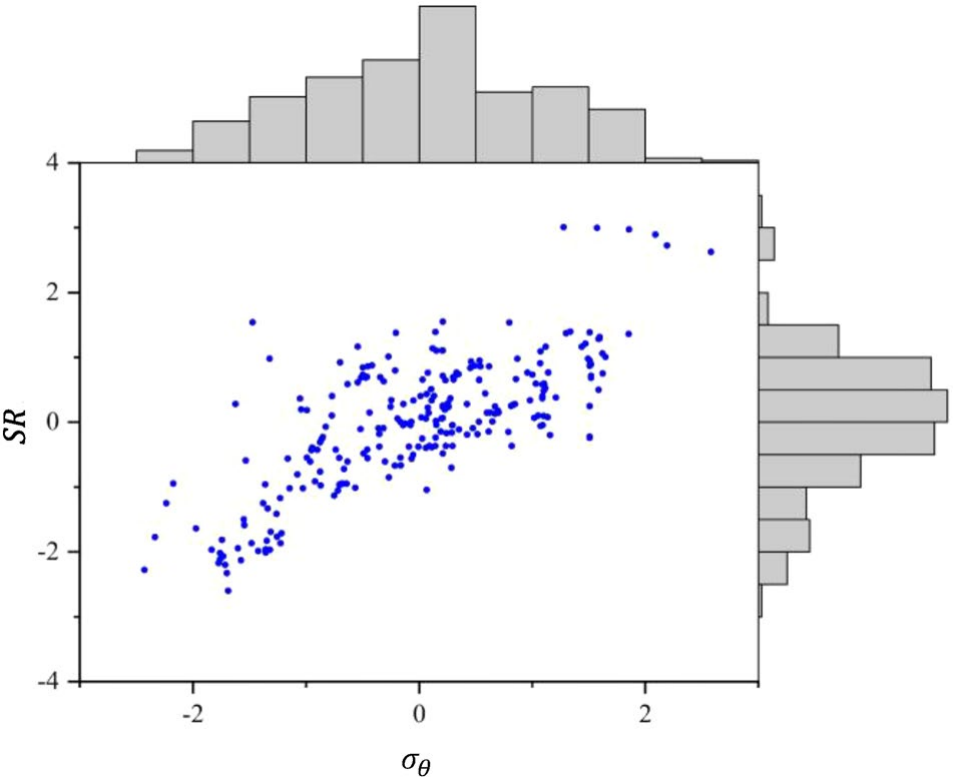
Data features after the Yeo–Johnson transformation.

High-dimensional digital features are difficult to display intuitively in space. Therefore, to illustrate the effect of the transformation, feature $\sigma_{\theta }$ with a smaller coefficient of variation and feature *SR* with a larger coefficient of variation are selected to construct the scatter plots in Figs. 3 and 4. Figure 3 shows the original data without scaling, whereas Fig. 4 shows the data after the Yeo–Johnson transformation. Surrounding the scatter plot is the marginal distribution of the corresponding features. From Figs. 3 and 4, it can be seen that the Yeo–Johnson transformation can reduce the gap between the clustered area with a large number of samples and the scattered area with a small number of samples. It also makes the distribution of points in the clustered area more uniform and reduces the influence of outliers on the prediction model.

### Rockburst data balancing based on K-means SMOTE

The frequencies of different rockburst grades are quite different, resulting in unbalanced rockburst data. There are two main approaches to address data imbalance: data level (oversampling, undersampling, and mixed sampling) and algorithm level^35^. At the data level, sampling methods are used to increase or decrease various rockburst samples to balance the dataset. The algorithm-level approach employs algorithms that are not sensitive to unbalanced datasets, such as Extra Tree, random forest (RF), and CatBoost.

Insufficient attention has been paid to the records of rockburst cases in engineering practice. There are only 275 rockburst cases in our dataset, of which 33 rockburst cases are grade IV. The under-sampling method can easily cause the loss of useful information, which leads to a decrease in the model accuracy. Therefore, oversampling method is more suitable. Machine learning algorithms usually require as large a dataset as possible. Oversampling methods can be divided into random and informed methods to generate oversampled samples^36^. Randomly generated oversampling samples can easily destroy the data structure and result in model overfitting. Among the informed generation methods for oversampling, the SMOTE algorithm can avoid overfitting. However, it may introduce noise to the dataset^37^. The borderline-SMOTE^38^ algorithm divides the data into three types: safety, danger, and noise. Only a few dangerous samples are oversampled, and thus no noise data will be generated. However, the algorithm has weaknesses in dealing between the within-class imbalance. Between-class means that the imbalance of the data sample numbers between the minority class and the majority class. Within-class imbalance means that the imbalance of the distribution position or distribution density of the sample.

Therefore, the K-means SMOTE algorithm is proposed to oversample the rockburst dataset after the Yeo–Johnson transformation. The K-means SMOTE algorithm consists of three steps: clustering, filtering, and oversampling. The clustering step divides the rockburst data into k clusters using the K-means algorithm. The filtering step retains clusters with a high proportion of minority samples, and then synthesizes more minority samples in sparse clusters. The oversampling step performs SMOTE oversampling on the clusters with a low density of minority samples. The sparser the minority samples in the cluster are, the more minority samples will be added. The algorithm identifies a sparse sample area by calculating the average distance of minority samples in a cluster, and generates more samples in the sparse sample area, which reduces the within-class imbalance^39^.

The calculation steps for K-means clustering are as follows:

1. Suppose the input dataset is $D = \{ x_{1} ,\;x_{2} , \ldots ,\;x_{m} \}$, and the division of clusters is $C = \{ C_{1} ,\;C_{2} , \ldots ,\;C_{k} \}$. Randomly select *k* samples from dataset *D* as the initial *k* centroid vectors, $\{ \mu_{1} ,\;\mu_{2} , \ldots ,\;\mu_{k} \}$.
2. Calculate the distance $d_{ik} = \| {x_{i} - \mu_{k} } \|_{2}^{2}$ between all sample points, *x*_*i*_, and each centroid vector, $\mu_{k}$; divide the sample points into the nearest cluster, $x_{i} \in C_{nearest}$; and update the cluster, $C_{nearest} = C_{nearest} \cup \{ x_{i} \}$.
3. Recalculate all sample points in cluster $C_{i}$; the new centroid is $\mu_{j} = \frac{1}{{| {C_{i} } |}}\sum\nolimits_{{x \in C_{i} }} x$.
4. Repeat calculation steps (2) and (3) until all of the centroid vectors, $\mu_{k}$, remain constant; output *C*.

The filtering step selects clusters with a high proportion of minority samples.

The oversampling step is performed as follows:

1. For each filtered cluster, $C_{i}$, calculate the Euclidean distance matrix, ignoring the majority samples.
2. Compute the mean distance, $d(C_{i} )$*,* within each cluster by summing all non-diagonal elements of the distance matrix, and then dividing by the number of non-diagonal elements.
3. Compute the density of each filtered cluster as $density(C_{i} ) = \frac{{mc(C_{i} )}}{{d(C_{i} )^{m} }}$, where $mc(C_{i} )$ is the number of minority samples in the cluster, and *m* is the number of features.
4. Calculate the sparsity of each filtered cluster as $sparsity(C_{i} ) = \frac{1}{{density(C_{i} )}}$.
5. Calculate the weight of each filtered cluster as $r(C_{i} ) = \frac{{sparsity(C_{i} )}}{{\sum\nolimits_{i = 1}^{k} {sparsity(C_{i} )} }}$.
6. Perform SMOTE oversampling for each filtered cluster. New samples are generated by interpolation from the minority samples in the cluster: $\vec{x} = \vec{a} + w \times (\vec{b} - \vec{a})$. In the filtered clusters, based on the sparseness of the minority samples, generate $r(C_{k} ) \times m$ new samples, where $\vec{x}$ is a newly generated sample, $\vec{a}$ is a randomly selected minority sample in the cluster, $\vec{b}$ is the nearest neighbor minority sample of $\vec{a}$, and *m* is the total number of samples in dataset *D*.

To compare the sensitivities of different algorithms to imbalanced datasets and the generalization abilities of various algorithms, the prediction results of 13 machine learning algorithms are compared for both the original rockburst dataset and rockburst dataset preprocessed by the Yeo–Johnson transform and K-Means SMOTE oversampling. The 13 machine learning algorithms considered are the support vector classifier (SVC), decision tree (DT), K-nearest neighbor (KNN), naive Bayes classifier (NBM), Gaussian processes (GP), multi-layer perceptron (MLP), quadratic discriminant analysis (QDA), random forest (RF), gradient boosting (GB), extreme gradient boosting (XgBoost), light boosting (LightBoost), extra tree (ET), and CatBoost. The accuracy, precision, recall rate, and F1 values are obtained for the training set and test set prediction results for each rockburst grade. Table 3 lists the prediction results obtained with the original rockburst dataset. Table 4 lists the prediction results obtained with the rockburst dataset after preprocessing. Stratified sampling of the dataset is used to divide the training and test sets such that the proportion of rockburst samples of each grade is consistent in the training and test sets. Three-quarters of the dataset is used as the training set to train the model, and the remaining 1/4 is used as the test set to evaluate the reliability and generalization ability of the model. In the model training process, grid search with cross-validation is used to obtain the optimal parameters with the highest accuracy.

**Table 3:** Prediction results with the original rockburst dataset.

Model	Rockburst grades	Training set				Test set			
		Precision	Recall rate	F1	Accuracy	Precision	Recall rate	F1	Accuracy
SVC	I	0.7353	0.6579	0.6944	0.6262	0.6667	0.7692	0.7143	0.5507
	II	0.5854	0.4364	0.5000		0.4167	0.2632	0.3226	
	III	0.6107	0.9091	0.7306		0.5476	0.7931	0.6479	
	IV	0.0000	0.0000	0.0000		0.0000	0.0000	0.0000	
DT	I	1.0000	1.0000	1.0000	1.0000	0.7273	0.6154	0.6667	0.6667
	II	1.0000	1.0000	1.0000		0.6842	0.6842	0.6842	
	III	1.0000	1.0000	1.0000		0.6875	0.7586	0.7213	
	IV	1.0000	1.0000	1.0000		0.4286	0.3750	0.4000	
KNN	I	1.0000	1.0000	1.0000	1.0000	0.8182	0.6923	0.7500	0.6377
	II	1.0000	1.0000	1.0000		0.6667	0.6316	0.6486	
	III	1.0000	1.0000	1.0000		0.6207	0.6207	0.6207	
	IV	1.0000	1.0000	1.0000		0.4545	0.6250	0.5363	
NBM	I	0.5952	0.6579	0.6250	0.5680	0.5000	0.7692	0.6061	0.4493
	II	0.5250	0.3818	0.4421		0.3077	0.2105	0.2500	
	III	0.6058	0.7159	0.6562		0.5457	0.5172	0.5263	
	IV	0.4000	0.3200	0.3556		0.2500	0.2500	0.2500	
GP	I	0.7500	0.7105	0.7297	0.7524	0.5714	0.6154	0.5926	0.4928
	II	0.6809	0.5818	0.6275		0.2308	0.1579	0.1875	
	III	0.7727	0.9659	0.8586		0.5385	0.7241	0.6176	
	IV	0.8462	0.4400	0.5789		0.6667	0.2500	0.3636	
MLP	I	0.7812	0.6579	0.7143	0.6942	0.7500	0.4615	0.5714	0.5217
	II	0.6304	0.5273	0.5743		0.4118	0.3684	0.3889	
	III	0.7182	0.8977	0.7980		0.5500	0.7586	0.6377	
	IV	0.5556	0.4000	0.4651		0.2500	0.1250	0.1667	
QDA	I	0.7742	0.6316	0.6957	0.6117	0.7143	0.7692	0.7407	0.5362
	II	0.5660	0.5455	0.5556		0.4375	0.3684	0.4000	
	III	0.6747	0.6364	0.6550		0.5926	0.5517	0.5714	
	IV	0.4103	0.6400	0.5000		0.3333	0.5000	0.4000	
GB	I	1.0000	1.0000	1.0000	1.0000	0.6667	0.6154	0.6400	0.6377
	II	1.0000	1.0000	1.0000		0.6000	0.6316	0.6154	
	III	1.0000	1.0000	1.0000		0.6774	0.7241	0.7000	
	IV	1.0000	1.0000	1.0000		0.5000	0.3750	0.4286	
XgBoost	I	1.0000	1.0000	1.0000	1.0000	0.7692	0.7692	0.7692	0.6522
	II	1.0000	1.0000	1.0000		0.6429	0.4737	0.5455	
	III	1.0000	1.0000	1.0000		0.6875	0.7586	0.7213	
	IV	1.0000	1.0000	1.0000		0.4000	0.5000	0.4444	
LightBoost	I	1.0000	1.0000	1.0000	0.9951	0.6923	0.6923	0.6923	0.6377
	II	1.0000	1.0000	1.0000		0.6000	0.4737	0.5294	
	III	1.0000	0.9886	0.9943		0.6471	0.7586	0.6984	
	IV	0.9615	1.0000	0.9804		0.5714	0.5000	0.5333	
RF	I	1.0000	1.0000	1.0000	1.0000	0.8182	0.6923	0.7500	0.6957
	II	1.0000	1.0000	1.0000		0.6316	0.6316	0.6316	
	III	1.0000	1.0000	1.0000		0.7097	0.7586	0.7333	
	IV	1.0000	1.0000	1.0000		0.6250	0.6250	0.6250	
ET	I	1.0000	1.0000	1.0000	1.0000	0.8000	0.6154	0.6957	0.6957
	II	1.0000	1.0000	1.0000		0.6316	0.6316	0.6316	
	III	1.0000	1.0000	1.0000		0.7188	0.7931	0.7541	
	IV	1.0000	1.0000	1.0000		0.6250	0.6250	0.6250	
CatBoost	I	1.0000	1.0000	1.0000	1.0000	0.7500	0.6923	0.7200	0.6957
	II	1.0000	1.0000	1.0000		0.7059	0.6316	0.6667	
	III	1.0000	1.0000	1.0000		0.6970	0.7931	0.7419	
	IV	1.0000	1.0000	1.0000		0.5714	0.5000	0.5333

**Table 4:** Prediction results with the rockburst dataset after preprocessing.

Model	Rockburst grades	Training set				Test set			
		Precision	Recall rate	F1	Accuracy	Precision	Recall rate	F1	Accuracy
SVC	I	0.9888	1.0000	0.9944	0.9802	0.9333	0.9333	0.9333	0.8051
	II	0.9888	0.9778	0.9832		0.7143	0.6667	0.6897	
	III	0.9767	0.9546	0.9655		0.6765	0.7931	0.7302	
	IV	0.9667	0.9886	0.9775		0.9231	0.8276	0.8727	
DT	I	1.0000	1.0000	1.0000	1.0000	0.8710	0.9000	0.8852	0.7966
	II	1.0000	1.0000	1.0000		0.6765	0.7677	0.7188	
	III	1.0000	1.0000	1.0000		0.7407	0.6897	0.7143	
	IV	1.0000	1.0000	1.0000		0.9231	0.8276	0.8727	
KNN	I	1.0000	1.0000	1.0000	1.0000	0.9355	0.9667	0.9508	0.8136
	II	1.0000	1.0000	1.0000		0.778	0.7000	0.7368	
	III	1.0000	1.0000	1.0000		0.7097	0.7586	0.7333	
	IV	1.0000	1.0000	1.0000		0.8276	0.8276	0.8276	
NBM	I	0.7300	0.8295	0.7766	0.6808	0.7714	0.9000	0.8308	0.6949
	II	0.6441	0.4222	0.5101		0.7059	0.4000	0.5105	
	III	0.5795	0.5795	0.5795		0.5714	0.6897	0.6250	
	IV	0.7383	0.8977	0.8103		0.7419	0.7931	0.7667	
GP	I	0.9556	0.9773	0.9663	0.9379	0.9333	0.9333	0.9333	0.7797
	II	0.9556	0.9556	0.9556		0.6667	0.6667	0.6667	
	III	0.9195	0.9091	0.9143		0.6562	0.7241	0.6885	
	IV	0.9295	0.9091	0.9143		0.8846	0.7931	0.8364	
MLP	I	1.0000	1.0000	1.0000	0.9972	0.8750	0.9333	0.9032	0.7627
	II	1.0000	1.0000	1.0000		0.6774	0.7000	0.6885	
	III	1.0000	0.9886	0.9943		0.6786	0.6552	0.6667	
	IV	0.9888	1.0000	0.9944		0.8148	0.7586	0.7857	
QDA	I	0.8049	0.7500	0.7765	0.6780	0.7586	0.7333	0.7458	0.6356
	II	0.6377	0.4889	0.5535		0.5833	0.4667	0.5185	
	III	0.5568	0.5568	0.5568		0.4722	0.5862	0.5231	
	IV	0.7043	0.9205	0.7980		0.7586	0.7586	0.7586	
GB	I	1.0000	1.0000	1.0000	1.0000	0.9032	0.9333	0.9180	0.7881
	II	1.0000	1.0000	1.0000		0.8400	0.7000	0.7636	
	III	1.0000	1.0000	1.0000		0.6364	0.7241	0.6774	
	IV	1.0000	1.0000	1.0000		0.7931	0.7931	0.7931	
XgBoost	I	1.0000	1.0000	1.0000	1.0000	0.9333	0.9333	0.9333	0.7797
	II	1.0000	1.0000	1.0000		0.7692	0.6667	0.7143	
	III	1.0000	1.0000	1.0000		0.6111	0.7586	0.6769	
	IV	1.0000	1.0000	1.0000		0.8462	0.7586	0.8000	
LightBoost	I	1.0000	1.0000	1.0000	0.9972	0.9655	0.9333	0.9492	0.7797
	II	1.0000	1.0000	1.0000		0.7857	0.7333	0.7586	
	III	1.0000	0.9886	0.9943		0.5938	0.6552	0.6230	
	IV	0.9888	1.0000	0.9944		0.7931	0.7931	0.7931	
RF	I	1.0000	1.0000	1.0000	1.0000	0.9333	0.9333	0.9333	0.7966
	II	1.0000	1.0000	1.0000		0.7500	0.7000	0.7241	
	III	1.0000	1.0000	1.0000		0.6667	0.7586	0.7079	
	IV	1.0000	1.0000	1.0000		0.8519	0.7931	0.8214	
ET	I	1.0000	1.0000	1.0000	1.0000	0.9355	0.9667	0.9508	0.8136
	II	1.0000	1.0000	1.0000		0.7586	0.7333	0.7458	
	III	1.0000	1.0000	1.0000		0.6667	0.7586	0.7097	
	IV	1.0000	1.0000	1.0000		0.9200	0.7931	0.8519	
CatBoost	I	1.0000	1.0000	1.0000	1.0000	0.9062	0.9667	0.9355	0.7966
	II	1.0000	1.0000	1.0000		0.8400	0.7000	0.7636	
	III	1.0000	1.0000	1.0000		0.6364	0.7241	0.6774	
	IV	1.0000	1.0000	1.0000		0.8214	0.7931	0.8070	

As can be seen from Tables 3 and 4, the test set of the original rockburst dataset has average precision, recall rate, and F1 values for grade I rockbursts of 0.7112, 0.6745, and 0.6855, respectively. The average precision, recall rate, and F1 values for the grade II rockbursts are 0.5443, 0.4737, and 0.5002, respectively. The average precision, recall rate, and F1 values for the grade III rockbursts are 0.6323, 0.7138, and 0.6686, respectively. The average precision, recall rate, and F1 values for the grade IV rockbursts are 0.4366, 0.4038, and 0.4082, respectively. The test set of the preprocessed rockburst dataset has average precision, recall rate, and F1 values for grade I rockbursts of 0.8965, 0.9205, and 0.9156, respectively. The average precision, recall rate, and F1 values of the grade II rockbursts are 0.7344, 0.6616, and 0.6923, respectively. The average precision, recall rate, and F1 values of the grade III rockbursts are 0.6397, 0.7135, and 0.6733, respectively. The average precision, recall rate, and F1 values of the grade IV rockbursts are 0.8384, 0.7931, and 0.8144, respectively. The results show that the rockburst dataset without data preprocessing has poor overall prediction results. In particular, the prediction results are lowest for the most hazardous grade IV rockbursts. After the Yeo–Johnson transformation and K-means SMOTE oversampling, the data structure is significantly improved, and a large number of outliers and data imbalance problems in the datasets for each rockburst grade are effectively addressed, thus improving the generalization ability of the model.

## Rockburst data feature analysis and determination of the optimal rockburst feature dimensions

### Rockburst data feature analysis

Breiman^40^ noted that an improvement in accuracy requires a more complex prediction model. It is usually difficult to achieve the best prediction accuracy using simple and interpretable models. However, complex machine learning algorithms inevitably have black box properties. To provide complex black box models with some interpretability, it is convenient to analyze the role of each feature in the prediction process.

**Figure 5:**
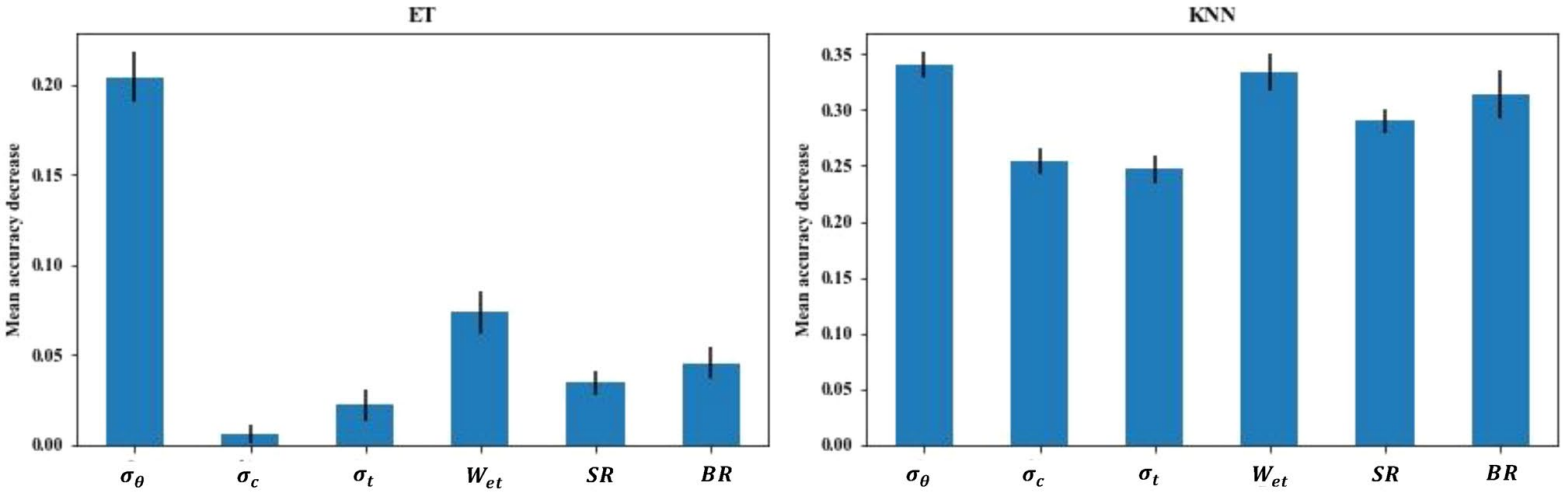
The mean decrease accuracy graph of ET and KNN models.

The ET and KNN models with the highest accuracy are used to evaluate the importance of features, and the importance of features is measured by the method of mean decrease accuracy. The method of reducing the average accuracy rate directly measures the impact of each feature on the accuracy of the model, by disrupting the order of the feature values of each feature, and measuring the impact of sequence changes on the accuracy of the model. For unimportant features, shuffling the order has little effect on the accuracy of the model. But for important features, disrupting the order will significantly reduce the accuracy of the model. Figure 5 shows the degree of mean decrease accuracy of the ET and KNN models, and the lines indicate the fluctuation range of the error.

In Fig. 5, the mean decrease accuracy of ET model features $\sigma_{\theta }$, $\sigma_{c}$, $\sigma_{t}$, $W_{et}$, *SR*, *BR* are respectively: 0.2044, 0.0061, 0.0222, 0.0739, 0.0345, 0.0458. The average accuracy reduction of KNN model features $\sigma_{\theta }$, $\sigma_{c}$, $\sigma_{t}$, $W_{et}$, *SR*, *BR* are respectively: 0.3398, 0.2538, 0.2470, 0.3339, 0.2898, 0.3144. Only feature $\sigma_{\theta }$ have a greater impact on ET model, while each feature has a greater impact on KNN model. This shows that different models have different degrees of dependence on features, and the role of features in the model will be quite different.

Correlation analysis of the features is performed to calculate the degree of correlation between two variables and analyze the degree of information redundancy contained in rockburst features^41^. Completely correlated variables represent truly redundant information, and adding completely correlated variables will not introduce additional information. Therefore, most scholars believe that redundant information contained in rockburst features will lead to poor model prediction results^42,43^. The PCA dimensionality reduction method is used to eliminate the correlations between rockburst features, which can eliminate features that contain less information. The Pearson correlation coefficient evaluates the linear relationship between two variables as follows:

2 $$ r = \frac{{N\sum {x_{i} y_{i} - \sum {x_{i} \sum {y_{i} } } } }}{{\sqrt {N\sum {x_{i}^{2} } - \left(\sum {x_{i} } \right)^{2} } \sqrt {N\sum {y_{i}^{2} } - \left(\sum {y_{i} } \right)^{2} } }} $$

where *x*_*i*_ is the *i*-th sample value of a certain rockburst feature, *y*_*i*_ is the *i*-th sample value of another rockburst feature, and *N* is the total number of samples.

**Figure 6:**
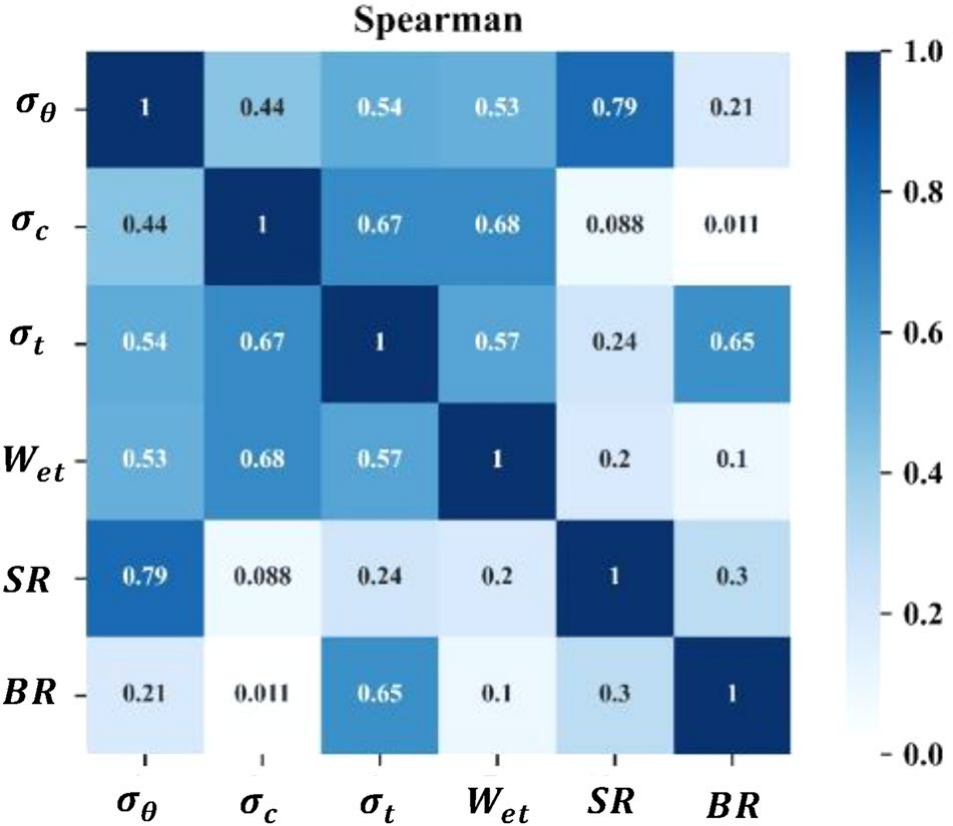
Heat map of the Pearson correlation coefficients of rockburst features.

The feature Pearson correlation coefficients are calculated for the preprocessed rockburst dataset, and the results are shown in Fig. 6. In general, if the absolute value of the Pearson's correlation coefficient is within 0.8–1.0, the two variables are considered very strongly correlated; if the Pearson’s correlation coefficient is within 0.6–0.8, 0.4–0.6, 0.2–0.4, and 0–0.2, the two variables are considered strongly correlated, moderately correlated, weakly correlated, and very weakly/not correlated, respectively. Figure 6 shows that there are no extremely strong correlations between rockburst features. There are strong correlations between $\sigma_{\theta }$ and *SR*, $\sigma_{c}$ and $\sigma_{t}$, $\sigma_{c}$ and $W_{et}$, and $\sigma_{t}$ and *BR*. Therefore, the rockburst features have partial information redundancy. In general, there is not an excessive amount of redundancy in the rockburst dataset, and each feature carries some unique information.

### Determination of the optimal rockburst feature dimension

In engineering practice, there are many factors affecting rockbursts, and there are more than ten corresponding indexes. However, in engineering practice, the record of rockburst cases has not received sufficient attention or it is difficult to obtain some features, which leads to a lack of some rockburst indicators in the available rockburst statistics, such as the point load strength of rock ($I_{s}$), deformation before the peak strength of rock ($U$), and stiffness of the loading process on the stress–strain curve ($K_{m}$). Relying only on six rockburst indicators ($\sigma_{\theta }$, $\sigma_{c}$, $\sigma_{t}$, $W_{et}$, *SR*, and *BR*) may have problems that cannot fully reflect the rockburst phenomenon. In general, before the curse of dimensionality, the more features are considered, the easier it is for the decision boundaries of the model to distinguish different categories, and the better the classification effect will be. If all features are predictive to a certain extent and the features are not completely correlated, then an appropriate increase in the number of features can improve the prediction ability^44,45^. Vong et al.^46^ noted that when the classified features resemble a family structure, the dataset will have a certain immunity to the curse of dimensionality, and an appropriate increase in the number of features is beneficial. However, the curse of dimensionality problem occurs when the data are high-dimensional^47^. It affects the learning process and reduces the accuracy.

To determine whether the rockburst dataset follows a family structure and the optimal number of classification features, first, on the basis of the six rockburst features, the PolynomialFeatures function in Sklearn is used to generate 21 new polynomial features. The method used to generate 21 new features is $\{ {a,\;b,\;a^{2} ,\;ab,\;b^{2} } \}$, where *a* is any feature of the original rockburst features, *b* is another arbitrary feature of the original rockburst features, and *a*^2^, *ab*, and *b*^2^ are newly generated features. Second, new features are generated from the original rockburst features, and these features inevitably have a strong correlation and excessive redundant information. Hence, PCA dimensionality reduction is used to process these 27 features, and the 27 principal components after PCA processing are retained. Finally, according to the amount of information contained in the principal components, principal components with less information are sequentially eliminated, and 26 rockburst datasets are constructed. These rockburst datasets contain two principal components, three principal components, etc., up to 27 principal components. Thirteen machine learning algorithms, SVC, DT, KNN, NBM, GP, MLP, QDA, RF, GB, XgBoost, LightBoost, ET, and CatBoost, are used to classify and predict the 26 rockburst datasets, resulting in a total of $26 \times 13 = 338$ classification prediction models.

PCA has three main functions. (1) When the number of samples is fixed and the features of the samples increase, the spatial distribution of samples becomes increasingly sparse, which leads to model overfitting. The PCA algorithm increases the sample density by discarding part of the information and alleviates the curse of dimensionality. (2) When the rockburst dataset is affected by noise, features with less information are often related to the noise, and eliminating features with little information can reduce noise. (3) In the rockburst dataset after PCA dimensionality reduction, each rockburst feature is independent of the others.

The PCA calculation steps are as follows:

1. Assume that the input dataset is $D = \{ x_{1}^{^{\prime}} ,\;x_{2}^{^{\prime}} , \ldots ,\;x_{m}^{^{\prime}} \}$, centralize each sample $x_{i}^{^{\prime}}$, and replace the original data with the centralized data as $x_{i} = x_{i}^{^{\prime}} - \frac{1}{m}\sum\nolimits_{j = 1}^{m} {x_{j}^{^{\prime}} }$.
2. Calculate the covariance matrix of the sample, $cov(x_{i} ,x_{j} )$, with $i,\;j = 1,\;2, \ldots ,\;m$.
3. Use the eigenvalue decomposition method to obtain the eigenvalues and eigenvectors of the covariance matrix.
4. Sort the eigenvalues from large to small, select the eigenvectors corresponding to the *k* largest eigenvalues, and normalize the eigenvectors to create the eigenvector matrix *W*.
5. Convert each sample $x_{i}$ to a new sample $z_{i} = W^{T} x_{i}$ and then obtain the output dataset, $D^{\prime} = (z_{1} ,\;z_{2} , \ldots ,\;z_{m} ).$

**Figure 7:**
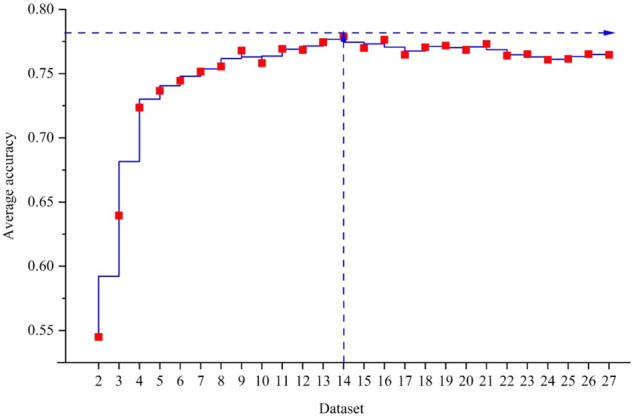
Average prediction accuracy of 26 datasets.

Because the classification performance of the 13 machine learning models is inconsistent, the model prediction ability will be the best for different numbers of principal components. Moreover, when using the stacking algorithm to integrate multiple models, the number of principal components must be consistent for all models. The quality of the model and the generalization ability are reflected in the test set. Therefore, the average prediction accuracy of 13 models in 26 datasets for the statistical test set is used as the basis for determining the optimal number of classification features. The results are shown in Fig. 7. In this figure, the numbers 2 to 27 on the abscissa represent datasets containing 2 to 27 principal components, and the ordinate represents the average prediction accuracy of the 13 models for the test set.

As shown in Fig. 7, the dataset with 14 retained principal components had the highest average prediction accuracy of 0.7790. The prediction accuracy rates of SVC, DT, KNN, NBM, GP, MLP, QDA, GB, XgBoost, LightBoost, RF, ET, and CatBoost for the test set were 0.7034, 0.7627, 0.8220, 0.6525, 0.7797, 0.7627, 0.7373, 0.8136, 0.8305, 0.8136, 0.8220, 0.8220, and 0.8051, respectively. Among them, the XgBoost model (0.8305) had the highest prediction accuracy, which was higher than that of the ET and KNN models (0.8136) that had the highest accuracy for the dataset after prediction processing (Table 4). To illustrate the relationship between the average prediction accuracy and the number of retained principal components, Fig. 7 shows two auxiliary lines with dashed arrows. In this figure, the accuracy with less than 14 retained principal components exhibits a fluctuating and gradually increasing trend, and the accuracy with greater than 14 retained principal components exhibits a fluctuating gradual decline. An appropriate increase in the number of independent principal components can improve the accuracy of rockburst prediction, and the rockburst dataset has certain characteristics of mitigating dimensional problems. This shows that the rockburst dataset conforms to the family structure described by Vong et al.^46^, and an appropriate increase in the number of rockburst features can improve the rockburst prediction.

## Rockburst prediction based on ensemble stacking

After the original rockburst dataset has undergone the Yeo–Johnson transformation, K-means SMOTE oversampling, and rockburst feature combinations to derive new features, the learning capabilities of the 13 machine learning models are improved to varying degrees. To further improve the accuracy of the rockburst prediction, stacking technology in ensemble learning is used to combine multiple machine learning methods to improve the model learning performance^48^. The ensemble stacking is divided into two layers. The first layer is fitted with multiple base models to output new features. The second layer uses the output of the first layer as the input. This stacking method for combining multiple learners is a type of meta-learning, which means learning to learn.

**Figure 8:**
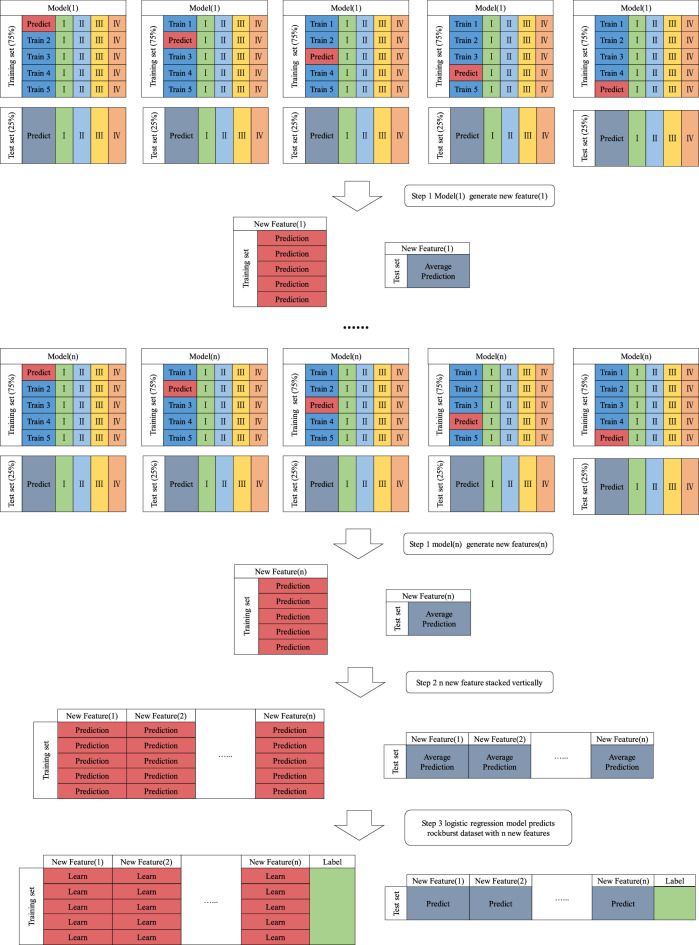
Ensemble stacking flow chart.

The stacking calculation process is divided into three steps, and the flowchart is shown in Fig. 8.

Step 1: First, select *n* machine learning models as the base model. The dataset with 14 rockburst features is divided into a training set (75%) and a test set (25%), and the training set is divided into five parts that are not crossed. Second, one of *Train 1*, *Train 2*, *Train 3*, *Train 4*, and *Train 5* in the training set is used as the validation set, and the remaining four datasets are used as the training set. Third, the base model performs five-fold cross-validation training on 75% of the training set and makes predictions based on the test set. Therefore, each set of *Train* data in the training set has a corresponding *Predict* value. Finally, each set of *Train* data in the training set is stacked, and new features generated by the base model are obtained from the training set; *n* base models generate *n* new features.

Step 2: Stack the *n* new features generated in Step 1 vertically for the training set and test set to obtain a new rockburst dataset.

Step 3: To prevent model overfitting, a logistic regression learner is used to train and predict the rockburst dataset with new features.

**Figure 9:**
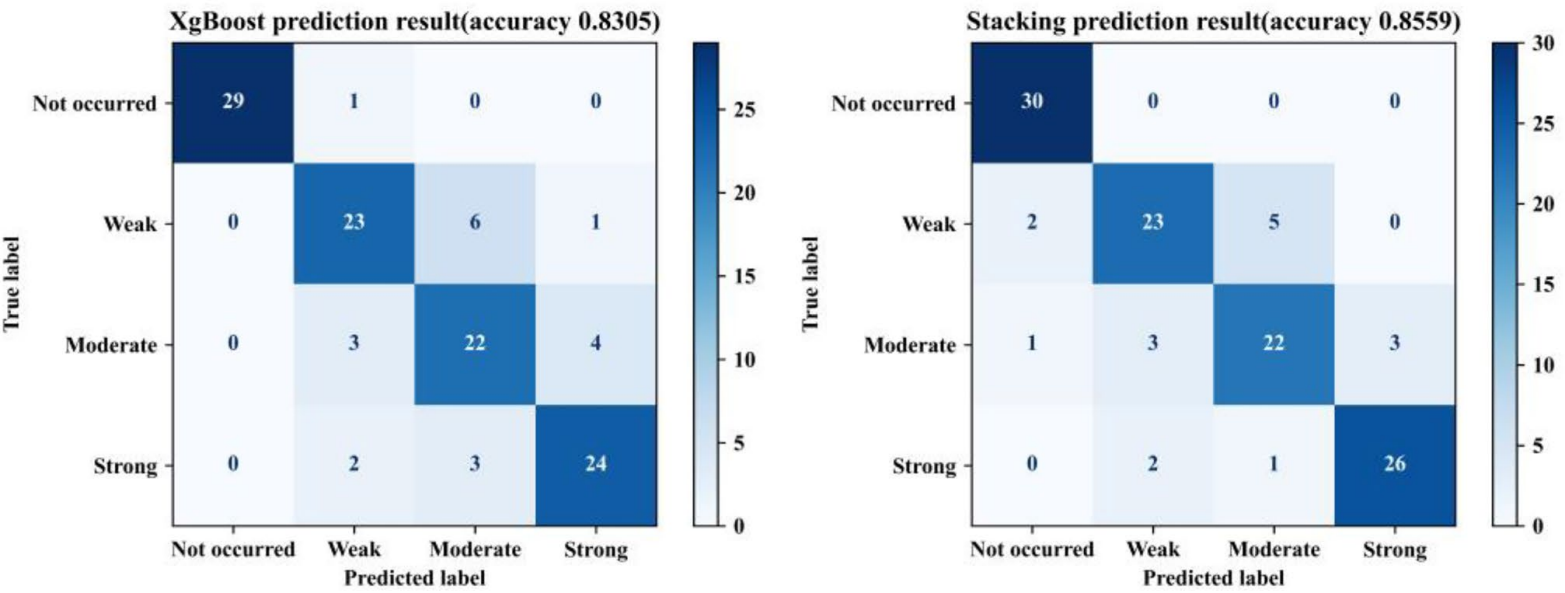
Confusion matrix diagrams for the XgBoost model and stacking model.

The XgBoost model has the highest accuracy in section “Determination of the optimal rockburst feature dimension”. Therefore, to demonstrate the advantages of rockburst prediction based on stacking, the confusion matrixes of the XgBoost model and stacking model for the test set are shown in Fig. 9. The abscissa in this figure represents the predicted result for each rockburst grade, and the ordinate represents the true result for each rockburst grade. The diagonal position of the XgBoost model confusion matrix shows that the correct prediction numbers for no rockburst, weak rockburst, moderate rockburst, and strong rockburst events are 29, 23, 22, and 24, respectively. The diagonal position of the stacking model confusion matrix shows that the correct prediction numbers for no rockburst, weak rockburst, moderate rockburst, and strong rockburst events are 30, 23, 22, and 26, respectively. The results show that the stacking model has a stronger generalization ability for no rockbursts and strong rockbursts, and its accuracy is higher than that of the highest accuracy XgBoost model.

## Conclusion

1. Using literature review methods, 275 sets of domestic rockburst data are collected to construct the original rockburst dataset. The proportion of each grade of rockburst, overlaid histograms of each feature in the rockburst dataset, and statistical parameters of different rockburst grades show that there are outliers and data imbalance phenomena in the dataset. The sparse outlier points are far from the dense point area. The ratio of the rockburst samples of grades I, II, III, and IV is 1.8:2.7:4.3:1.2, and the rockburst samples in each grade are unbalanced.
2. To address the phenomena of outliers and data imbalance in the rockburst dataset, the Yeo–Johnson transformation is proposed to normalize the data distribution and reduce the interval between outliers and the cluster area, thereby reducing the impact of outliers on the forecast results. The K-means SMOTE algorithm is used to oversample the rockburst data set after the Yeo–Johnson transformation to ensure the rockburst samples attain both within-class balance and between-class balance. After data processing through the Yeo–Johnson transform and K-means SMOTE oversampling, the prediction accuracy of 13 single machine learning algorithm models is increased by an average of 0.1638.
3. Rockburst data has a family resemblance structure. Therefore, an appropriate increase in the number of features can improve or maintain the prediction ability. A method of multiplying two-by-two based on six original features and squaring a single original feature is adopted to generate 21 new features and construct a dataset with 27 rockburst features. Then, PCA technology is used to eliminate the correlations between features, ensuring each feature is independent of the others. The exhaustive method selects the number of features that produces the highest average accuracy of the 13 machine learning algorithms, and the average accuracy of the rockburst dataset with 14 features is 0.7790.
4. After the Yeo–Johnson transformation, K-means SMOTE oversampling, and determination of the optimal rockburst feature dimension of the original rockburst dataset, the rockburst data structure is significantly improved. To further improve the accuracy of rockburst prediction, the prediction ability is improved at the algorithm level. Fourteen rockburst features are used as the input for stacking; multiple machine learning algorithms are used as the first-level base model, and a logistic regression classifier is used as the second-level output model. Compared with 13 single machine learning models optimized for the data structure, the ensemble stacking model has an average prediction accuracy improvement of 0.0769.

## Data Availability

All data that support the findings of this study are available from the corresponding author upon reasonable request.
